# Rattlesnake *Crotalus molossus nigrescens* venom induces oxidative stress on human erythrocytes

**DOI:** 10.1186/s40409-017-0114-y

**Published:** 2017-04-21

**Authors:** David Meléndez-Martínez, Juan Manuel Muñoz, Guillermo Barraza-Garza, Martha Sandra Cruz-Peréz, Ana Gatica-Colima, Emilio Alvarez-Parrilla, Luis Fernando Plenge-Tellechea

**Affiliations:** 1 Departamento de Ciencias Químico Biológicas, Instituto de Ciencias Biomédicas, Universidad Autónoma de Ciudad Juárez, Anillo Envolvente del PRONAF y Estocolmo s/n, C. P. 32310. A. P. 1595-D Ciudad Juárez, Chihuahua Mexico; 2 Herpetario de la Universidad Autónoma de Querétaro, Facultad de Ciencias Naturales, Universidad Autónoma de Querétaro, Juriquilla, Querétaro, Mexico

**Keywords:** Attenuated total reflectance-Fourier transform infrared spectroscopy, *Crotalus molossus nigrescens*, Venom, Snake venom, Methemoglobin, Oxidative stress, Oxyhemoglobin

## Abstract

**Background:**

Globally, snake envenomation is a well-known cause of death and morbidity. In many cases of snakebite, myonecrosis, dermonecrosis, hemorrhage and neurotoxicity are present. Some of these symptoms may be provoked by the envenomation itself, but others are secondary effects of the produced oxidative stress that enhances the damage produced by the venom toxins. The only oxidative stress effect known in blood is the change in oxidation number of Fe (from ferrous to ferric) in hemoglobin, generating methemoglobin but not in other macromolecules. Currently, the effects of the overproduction of methemoglobin derived from snake venom are not extensively recorded. Therefore, the present study aims to describe the oxidative stress induced by *Crotalus molossus nigrescens* venom using erythrocytes.

**Methods:**

Human erythrocytes were washed and incubated with different *Crotalus molossus nigrescens* venom concentrations (0–640 μg/mL). After 24 h, the hemolytic activity was measured followed by attenuated total reflectance-Fourier transform infrared spectroscopy, non-denaturing PAGE, conjugated diene and thiobarbituric acid reactive substances determination.

**Results:**

Low concentrations of venom (<10 μg/mL) generates oxyhemoglobin release by hemolysis, whereas higher concentrations produced a hemoglobin shift of valence, producing methemoglobin (>40 μg/mL). This substance is not degraded by proteases present in the venom. By infrared spectroscopy, starting in 80 μg/mL, we observed changes in bands that are associated with protein damage (1660 and 1540 cm^−1^) and lipid peroxidation (2960, 2920 and 1740 cm^−1^). Lipid peroxidation was confirmed by conjugated diene and thiobarbituric acid reactive substance determination, in which differences were observed between the control and erythrocytes treated with venom.

**Conclusions:**

*Crotalus molossus nigrescens* venom provokes hemolysis and oxidative stress, which induces methemoglobin formation, loss of protein structure and lipid peroxidation.

## Background

Snakebites cause considerable death and high morbidity worldwide and pose an important threat to public health, especially those provoked by *Crotalus* snakes [1–4]. Rattlesnake envenomation alters the homeostasis of victims, generating coagulation alterations and hemorrhage that may provoke death. Rattlesnake envenomation may cause myonecrosis, dermonecrosis, neurotixicity, different types of damage to the vascular extracellular matrix, and edema [5]. These effects are generated by some toxins such as phospholipases A_2_ (PLA_2_), low molecular mass myotoxins, L-amino acid oxidases (LAAO) and proteases including metalloproteinases (SVMPs) and serineproteinases (SVSPs) [6–8]. In addition, reactive oxygen species (ROS) are produced as a side effect of the catalytic activity of some toxins, enhancing the damage produced by the venom and causing systemic oxidative stress [9].

The different scenarios on the oxidative stress indicate that are unrecognized and underestimated forms of affliction provoked by snakes. SVMPs, major components in most Crotalid venoms, have a relevant role in venom-induced local damage. The lethality of this venom is due to the high activity of PLA_2_ that hydrolyzes phospholipids and releases arachidonic acid, which, in turn, generates toxic reactive oxygen species (ROS) [10, 11]. This reaction results in lipid peroxidation and leads to cellular damage [12].

Cells have antioxidant defenses against toxic ROS – such as the enzymes superoxide dismutase (SOD) and catalase (CAT) – that work in tandem to neutralize superoxide radicals [13–15]. SOD catalyzes the loss of superoxide radicals into oxygen or hydrogen peroxide (H_2_O_2_); this is itself detrimental and must be detoxified into other non-toxic substances. By another way, CAT helps in the decomposition of H_2_O_2_ into water and oxygen [16]. All these responses are part of the reactions triggered by *C. m. nigrescens* venom (CMNv), which produce significant oxidative stress in different stages. Thus, an important role of lipid peroxidation in cytotoxicity of *C. m. nigrescens* envenomation is suggested. Based on these relevant findings of oxidative stress produced by snakebite, the present study aims to describe the oxidative stress induced by CMNv using a model of isolated erythrocytes.

## Methods

### Venom

Venom samples were obtained from *C. m. nigrescens* specimens maintained at the Universidad Autónoma de Queretaro herpetarium. Venom extraction was performed manually as described by Meléndez-Martínez et al. [17]. CMNv was pooled, lyophilized and stored at −20 °C until use. Protein concentration in venom was measured by Lowry protein assay [18], using bovine serum albumin as standard.

### Hemolytic activity

Hemolytic activity was determined using Das et al. [19] protocol with some modifications. Blood was collected from at least three healthy donors for each experiment. Inclusion criteria were: O+ donors that had not taken any medication 48 h before the test. Blood samples were collected in BD Vacutainer® buffered sodium citrate tubes and erythrocytes were isolated and washed thrice by centrifugation at 1,100 g and re-suspended in 0.9% saline solution to a final concentration of 20%. Then, 37.5 μL of erythrocyte suspension was incubated during 24 h at 37 °C with different CMNv concentrations (0–640 μg/mL) in a 500 μL final volume. Next, the samples were centrifuged at 6,400 *g* for 10 min and supernatant was measured at 540 nm for oxyhemoglobin (Oxy-Hb) and 630 nm for methemoglobin (Met-Hb) in a Helios Omega UV–vis spectrophotometer (Thermo Scientific, USA). As 100% Oxy-Hb and Met-Hb controls distilled H_2_O and H_2_O_2_ 5% (v/v) were used, respectively.

### Polyacrylamide gel electrophoresis

These experiments were carried out under non-denaturing and denaturing polyacrylamide gel electrophoresis (PAGE) using a 15% acrylamide gel modified from Sambrook and Russell [20] to observe the degradation of the hemoglobin (Hb). Treated erythrocytes were centrifuged as described above and 50 μg of soluble Hb was collected to use in the gels and stained 0.1% Coomassie blue R-250 staining. The samples used in denaturing PAGE were sand boiled by 5 min before the electrophoresis.

### Attenuated total reflectance-Fourier transform infrared spectroscopy

Erythrocytes were treated as described in hemolysis assay. The CMNv treated erythrocytes were analyzed in attenuated total reflectance-Fourier transform infrared (ATR-FTIR) in a Bruker Alpha FTIR Spectrometer (Bruker Optics, USA), according to the method described in Barraza-Garza et al. [21]. Briefly, 4 μL of treated erythrocytes were placed at the spectrometer and were allowed to dry for 10 min. Spectra were recorded in a range from 4000 to 900 cm^−1^ with a maximum resolution of 6 cm^−1^, and 200 scans per spectrum were collected. Three spectra were recorded for each treatment.

### Infrared spectra analysis

Infrared (IR) spectra were analyzed using Unscrambler® X software (CAMO Software, Norway). Raw spectra were preprocessed using first vector normalization and then a second derivative using Savitsky-Golay of second polynomial order with 21 smoothing points. The second derivative spectra obtained was analyzed in order to observe differences in intensity and position of the signals. The analysis was made in three main zones across the spectra that are related to chemical bonds liable to damage via oxidative stress [21]: ~1550-1660 cm^−1^ region for amide I and II, ~1740-1780 cm^−1^ region for aldehyde bond and ~2920-2960 cm^−1^ region for primary and secondary carbon bonds in lipid skeletons.

### Conjugated dienes determination

Conjugated dienes (CD) were determined according to Barraza-Garza et al. [21] with slight modifications. After treatment, the erythrocytes were resuspended in 900 μL of methanol:chloroform (1:2) solution and vortexed for 2 min. The mixture was centrifuged for 10 min at 4650 *g* and the chloroform phase was collected and dried with a nitrogen flux. Then, lipids were dissolved in 200 μL of cyclohexane, mixed by vortex for 30 s and immediately measured at 233 nm in a Helios Omega UV–vis spectrophotometer (Thermo Scientific, USA) using cyclohexane as blank. Absorbance was converted to conjugated dienes concentration using an extinction coefficient of 27000 M^−1^cm^−1^ and the results were expressed as concentration (μM) of conjugated dienes per sample.

### Thiobarbituric acid reactive substances assay

Thiobarbituric acid reactive substances assay (TBARS) was performed according to Barraza-Garza et al. [21] with slight modifications. After treatment, 100 μL of venom-treated erythrocytes were mixed with 400 μL of thiobarbituric acid reagent (20% trichloroacetic acid; 0.5% thiobarbituric acid and 2.5 N HCl) and the mixture was heated for 1 h at 75 °C. After cooling, the solution was centrifuged at 380 *g* for 10 min and the absorbance of supernatant was measured at 532 nm in a Helios Omega UV–vis spectrophotometer (Thermo Scientific, USA) using the reaction mixture as a blank. Results were expressed as concentration (μM) of malondialdehyde (MDA) per sample. A calibration curve was prepared with tetramethoxypropane (which is equivalent to the same concentrations of MDA) with concentrations ranging from 1.25 to 20 μM.

### Statistical analysis

Results obtained with CD and TBARS were analyzed by a one-way analysis of variance (ANOVA). When ANOVA showed a significant difference, Tukey’s post hoc test was applied. Multivariate analysis was made by PCA as described in the previous subsection, using Unscrambler® X software (CAMO Software, Norway).

## Results and discussion

### Hemolytic activity

**Fig. 1:**
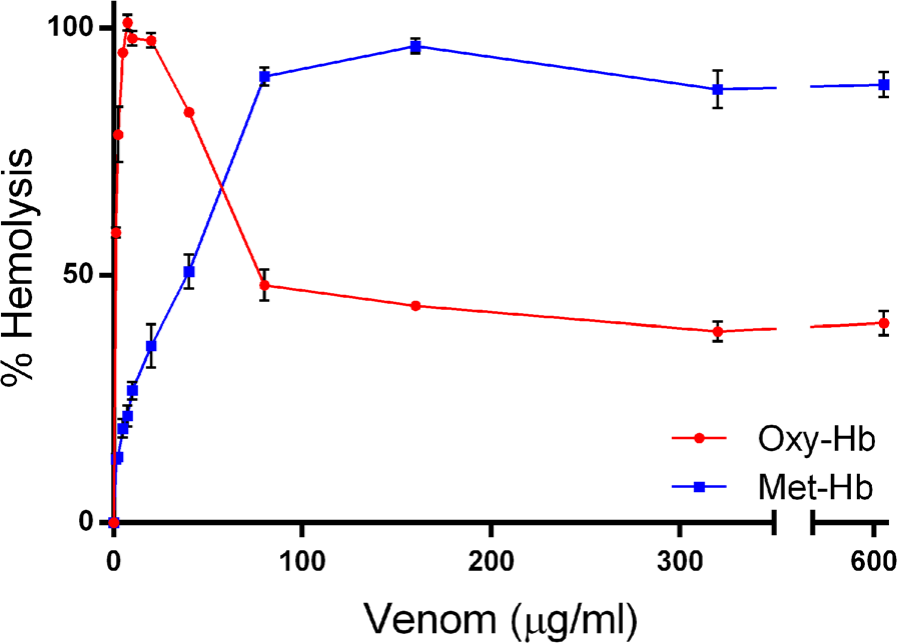
Hemolytic effect of CMNv. Erythrocytes were treated with different concentrations of CMNv (0–640 μg/mL) for 24 h. Oxy-Hb is represented by a red line and Met-Hb by a blue line. Data are represented as the mean of three independent experiments with its respective standard error

In this assays we found that CMNv produces hemolysis observed as an Oxy-Hb release from the lysed erythrocyte (Fig. 1), the median hemolytic concentration was 1.00 ± 0.07 μg/mL and showed a full hemolysis with 7.5 μg/mL of venom. At higher CMNv concentrations, > 40 μg/mL, the Oxy-Hb concentration decreased and Met-Hb increased in a concentration-dependent manner having its higher concentration (≈90%) at 160 μg/mL of venom.

Hemolysis could be produced as consequence of PLA_2_ phospholipid degradation and L-amino acid oxidase catalysis [22, 23]. Due to Oxy-Hb release, the ferrous (Fe^2+^) heme was converted to ferric (Fe^3+^) heme resulting in Met-Hb. This valence shift in Hb could be generated by oxidative stress induced by H_2_O_2_ produced by the LAAO catalysis [24, 25]. Then, Hb damage could be magnified by the Met-Hb formation itself, acting as a prooxidant molecule accelerating de process of Met-Hb formation [26].

### Hemoglobin degradation

**Fig. 2:**
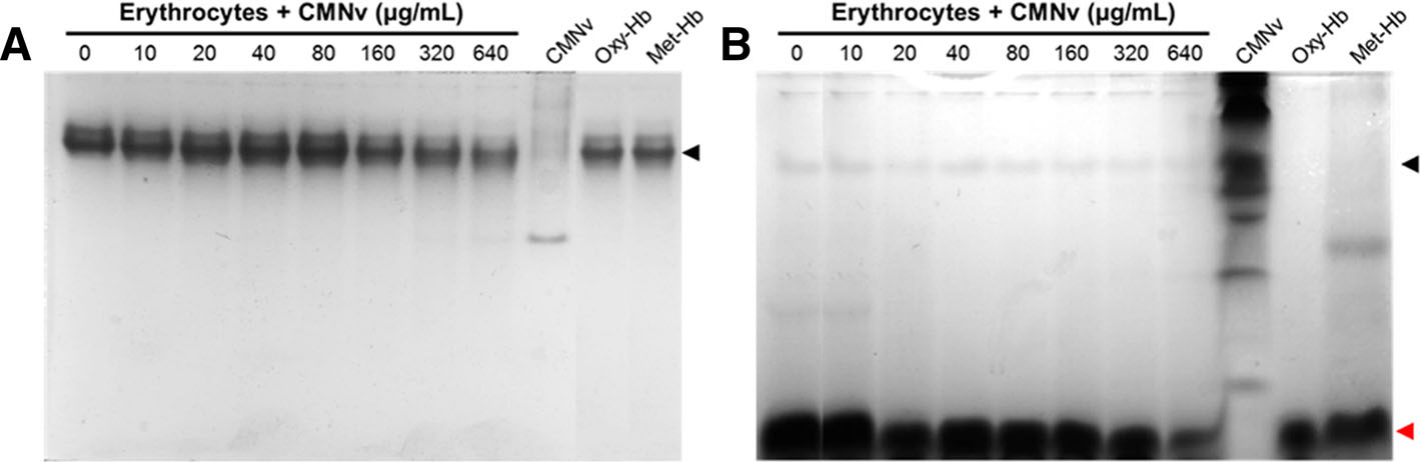
PAGE of hemoglobin under (**a**) non-denaturing and (**b**) denaturing conditions – erythrocytes incubated with CMNv. Erythrocytes treated with different concentrations of CMNv for 24 h were centrifuged and soluble hemoglobin was used in this experiment. Lanes 1–8 show the erythrocytes treated with different venom concentrations, lane 9 shows 50 μg of CMNv, lanes 10 and 11 show Oxy-Hb and Met-Hb controls, respectively. The hemoglobin (64 kDa) is marked with a black arrow, α-globin and β-globin (16 kDa) are marked with a red arrow head

The Hb exposed to the CMNv not only showed a valence shift, from Oxy-Hb to Met-Hb, but also a slight degradation of Met-Hb. The higher venom concentrations (160, 320 and 640 μg/mL) produced the Hb partial degradation (Fig. 2). Apparently, the proteases contained in the venom, SVMPs and SVSPs, had lesser affinity to Hb than other substrates as fibrinogen [27]. Moreover, basement membrane components including perlecan and nidogen are hydrolyzed within minutes, destabilizing the basement membrane and leading to hemorrhage [28, 29].

### Oxidative stress evaluation by attenuated total reflectance-Fourier transform infrared spectroscopy

Infrared spectroscopy allowed us to observe differences of the different biomolecules on erythrocytes due to oxidative stress by CMNv concentration increase. ATR-FTIR analysis was carried out at the regions associated to lipid (2960, 2920 and 1740 cm^−1^) and protein oxidation (1660 and 1540 cm^−1^) bands to determine the oxidation in proteins. No differences were observed between control samples and those treated with a low venom concentration (10–40 μg/mL).

**Fig. 3:**
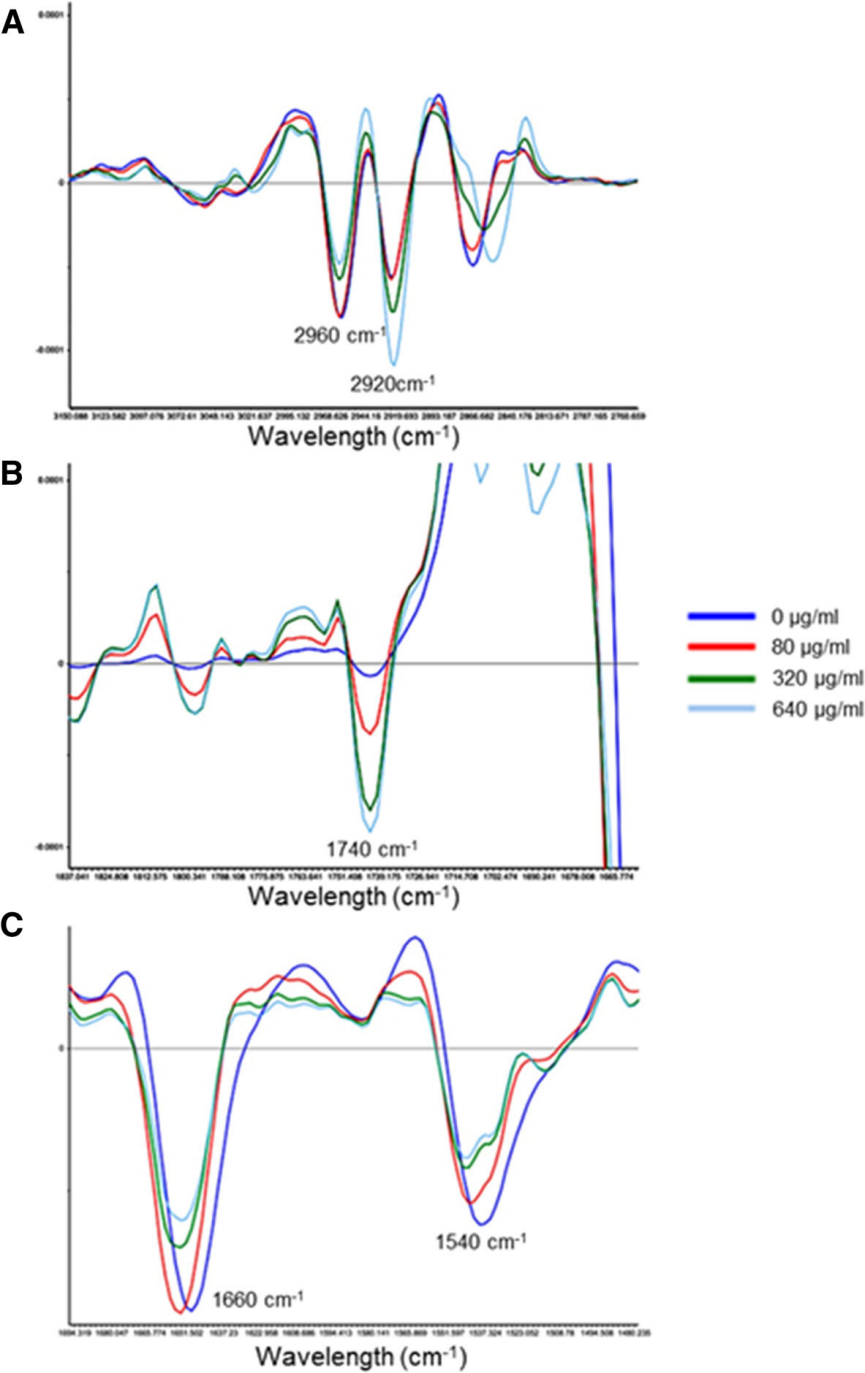
ATR-FTIR second derivative spectra of the erythrocytes incubated with different CMNv concentrations (0,80, 320 and 640 μg/mL). (**a**) Second derivative of skeletal carbons of lipids, primary carbon (2960 cm^−1^) and secondary carbon (2920 cm^−1^) bands. **b** Second derivative of aldehyde bond band (1740 cm^−1^) related to secondary products of lipid peroxidation. **c** Second derivative of the amide bonds region, amide I (1660 cm^−1^) and amide II (1540 cm^−1^) that are the main bands related to protein structure

Changes observed in the region from 2920 to 2960 cm^−1^ are related to primary and secondary carbons in the lipid skeletons, respectively (Fig. 3a). The 80 μg/mL treatment showed the same IR profile as control, were the primary carbon band (2920 cm^−1^) had a higher intensity than the secondary carbon band (2920 cm^−1^), meaning that the lipid structure is not modified. At higher venom concentrations (320 and 640 μg/mL) the proportion between primary and secondary carbons switches, showing higher intensity in the secondary carbon band; this shift may be related to the loss of integrity in the lipid structure. Benseny-Cases et al. [30] reported similar results when membrane lipids were attacked by oxidative radicals. They observed changes in the proportion of primary and secondary carbons in their skeletal lipids. In Fig. 3b, it can be seen that the peak intensity at 1740 cm^−1^ (associated to aldehyde compounds) increased as the venom concentration increased. Aldehyde compounds are secondary products of lipid oxidation, meaning that the venom may act as oxidative stress generator [21].

Changes in both amide I and II can be seen at venom concentrations higher than 80 μg/mL. In Fig. 3c it is possible to observe a loss of symmetry of the peak and a displacement to higher wave number, and a decrease of intensity of both bands as the concentration of venom increases. This decrease in the intensity of the bands can be associated with changes in the secondary and tertiary structure from proteins, mainly Hb, related to the loss of integrity of proteins caused by the oxidative stress [31].

### Spectrophotometric measures of lipid peroxidation by conjugated dienes and thiobarbituric acid reactive substances assay

In the IR spectra analysis we saw a clear variation in the lipid composition of the samples, as we had mainly two types of lipid damage by the venom, namely:

- a change in the proportion of primary and secondary carbons of the lipid skeleton related to structure loss in lipids by lipid peroxidation;
- an intensity increase of the aldehyde band related to the production of secondary products of the lipid peroxidation.

**Fig. 4:**
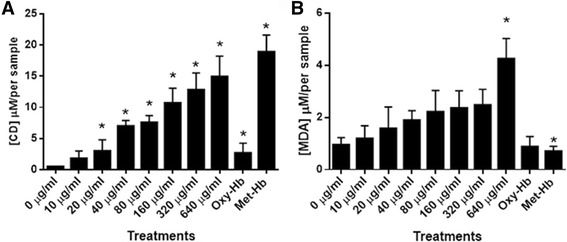
Lipid peroxidation measurement by (**a**) CD and (**b**) TBARS assays of the erythrocytes incubated with *Crotalus molossus nigrescens* venom. Data are represented as the mean of various independent experiments with its respective standard error, statistical significance difference (*p* < 0.05) respect to the control (0 μg/mL) is represented with an asterisk (*)

In order to confirm the oxidation observed by FTIR, two spectrophotometric assays (TBARS and CD) were carried out. In Fig. 4a, it is possible to observe that the amount of CD augmented steadily as the concentration of venom increased. Considering that CD is a primary product of lipid peroxidation, it is clear that CMNv lipid peroxidation products are generated by the oxidative stress provoked by the presence of the venom in a dose-dependent manner. In addition, we observed a low lipid peroxidation in Oxy-Hb control in comparison to Met-Hb control, demonstrating that the Met-Hb can act as a prooxidant molecule. Based on the present results, the lipid peroxidation effects seen in the IR analysis are corroborated.

TBARS assay validated the effects observed in the ATR-FTIR assay for aldehyde band (Fig. 4b) as well as for CD. Through TBARS assay, it was possible to observe an increase of MDA as the concentration of venom augmented. However, this increase was only significant for the highest venom concentration. Considering that TBARS represent the formation of secondary products of lipid peroxidation, this results may suggest that at the time and concentration studied, the venom produced mainly primary products of lipid oxidation. Interestingly, both Oxy-Hb and Met-Hb treatments had a lower concentration of MDA than that seen in the control treatment (0 μg/mL), suggesting that the catalytic activity of the venom is involved in the production of aldehyde compounds and not as an oxidative effect of Met-Hb.

This increment of MDA could be produced due the arachidonic acid release after PLA_2_ catalysis [11]. Moreover, the newly produced arachidonic acid could be oxidized by H_2_O_2_ produced by the LAAO catalysis [25]. Al Asmari et al. [32] reported an increment of MDA levels in the liver, kidney, heart, and brain of mice treated with *Echis pyramidum* venom. Similarly to our results, they reported a MDA increase after 24 h of incubation, suggesting that the oxidative stress produced by the CMNv in the erythrocytes can lead to a systemic lipid peroxidation affecting other tissues.

## Conclusions

In conclusion, through ATR-FTIR, CD, and TBARS we demonstrate that the venom of *Crotalus molossus nigrescens* is an oxidative stress inductor generating Met-Hb, loss of protein structure and lipid peroxidation in erythrocytes, which can be related to some of the symptoms observed in the envenomation.

## Abbreviations

ANOVA: One-way analysis of variance
ATR-FTIR: Attenuated total reflectance-Fourier transform infrared
CAT: Catalase
CD: Conjugated dienes
CMNv: *Crotalus molossus nigrescens* venom
H_2_O_2_: Hydrogen peroxide
Hb: Hemoglobin
IR: Infrared
LAAO: L-amino acid oxidases
MDA: Malondialdehyde
Met-Hb: Methemoglobin
Oxy-Hb: Oxyhemoglobin
PAGE: Polyacrylamide gel electrophopresis
PLA_2_: Phospholipase A_2_
ROS: Reactive oxygen species
SOD: Superoxide dismutase
SVMPs: Snake Venom Metalloproteinases
SVSPs: Snake Venom Serineproteinases
TBARS: Thiobarbituric acid reactive substances assay

## References

[1] Massougbodji M, Chobli M, Assouto P, Lokossou T, Sanoussi H, Sossou A (2002). Geoclimatology and severity of snake bite envenomations in Benin. Bull Soc Pathol Exot.

[2] Gutiérrez JM (2014). Current challenges for confronting the public health problem of snakebite envenoming in Central America. J Venom Anim Toxins incl Trop Dis.

[3] Habib GA (2013). Public health aspects of snakebite care in West Africa: perspectives from Nigeria. J Venom Anim Toxins incl Trop Dis.

[4] Chippaux JP (2015). Epidemiology of envenomations by terrestrial venomous animals in Brazil based on case reporting: from obvious facts to contingencies. J Venom Anim Toxins incl Trop Dis.

[5] Gutierrez JM, Lomonte B, Leon G, Rucavado A, Chaves F, Angulo Y (2007). Trends in snakebite envenomation therapy: scientific, technological and public health considerations. Curr Pharm Des.

[6] Calvete JJ, Fasoli E, Sanz E, Boschetti E, Righetti PG (2009). Exploring the venom proteome of the estern diamondback rattlesnake, *Crotalus atrox*, via snake venomics and combinational peptide ligand library approaches. J Proteome Res.

[7] Costa TR, Burin SM, Menaldo DL, Castro FA, Sampaio SV (2014). Snake venom L-amino acid oxidases: an overview on their antitumor effects. J Venom Anim Toxins incl Trop Dis.

[8] Al-Quraishy S, Dkhil MA, Moneim AEA (2014). Hepatotoxicity and oxidative stress induced by Naja haje crude venom. J Venom Anim Toxins incl Trop Dis.

[9] Sunitha K, Hemshekhar M, Thushara RM, Sebastin-Santhosh M, Shanmuga-Sundaram M, Kemparaju K, Girish KS (2015). Inflammation and oxidative stress in viper bite: An isight within and beyond. Toxicon.

[10] He W, Xiaole C, Mei Z, Lei W, Tianbao C, Chris S (2016). Molecular characterization of three novel phospholipase A2 proteins from the venom of atheris chlorechis, atheris nitschei and atheris squamigera. Toxins.

[11] Park MH, Jo M, Won D, Song HS, Song MJ, Hong JT (2012). Snake venom toxin from *Vipera lebetina turanica* sensitizes cancer cells to TRAIL through ROS- and JNK-mediated upregulation of death receptors and downregulation of survival proteins. Apoptosis.

[12] Nanda BL, Nataraju A, Rajesh R, Rangappa KS, Shekar MA, Vishwanath BS (2007). PLA2 mediated arachidonate free radicals: PLA2 inhibition and neutralization of free radicals by antioxidants--a new role as anti-inflammatory molecule. Curr Top Med Chem.

[13] Abdurrahman AA, Haseeb AK, Khalaf AM, Rajamohamed AM (2006). Time-course of lipid peroxidation in different organs of mice treated with *Echis pyramidum* snake venom. J Biochem Mol Toxicol.

[14] Barraviera B, Machado PEA, Meira DA, Curi PR, Martins JNP, Souza MJ (1988). Glucose-6-phosphate dehydrogenase and glutathione reductase activity in methemoglobin reduction by methylene blue and cystamine. Study on glucose-6-phosphate dehydrogenase-deficient individuals, on normal subjects and on riboflavin-treated subjects. Rev Inst Med Trop Sao Paulo.

[15] Barraviera B (1989). Effect of antimalarial drugs and of clindamycin on erythrocyte metabolism. A review. Rev Inst Med Trop Sao Paulo.

[16] Du XY, Clemetson KJ (2002). Snake venom L-amino acid axidases. Toxicon.

[17] Meléndez-Martínez D, Macias-Rodríguez E, Vargas-Caraveo A, Martínez-Martínez A, Gatica-Colima A, Plenge-Tellechea LF (2014). Capillary damage in the area postrema by venom of the northern black-tailed rattlesnake *(Crotalus molossus molossus*). J Venom Res.

[18] Lowry OH, Rosenbrough NJ, Farr AL, Randall RJ (1951). Protein measurement with the Folin Phenol Reagent. J Biol Chem.

[19] Das D, Urs N, Hiremath V, Vishwanath BS, Doley R (2013). Biochemical and biological characterization of Naja kaouthia venom from North-East India and its neutralization by polyvalent antivenom. J Venom Res.

[20] Sambrook J and Russell DW. Molecular cloning, A laboratory manual. 3rd ed. New York: Cold Spring Harbor laboratory Press; 2001.

[21] Barraza-Garza G, Castillo-Michel H, de la Rosa LA, Martinez-Martinez A, Perez-Leon JA, Cotte M, Alvarez-Parrilla E. Infrared Spectrocospy as a tool to study the antioxidant activity of polyphenolic compounds in isolated rat enterocytes. Oxid Med Cell Longev. 2016;1:1–10. 10.1155/2016/9245150 PMC4861801 27213031

[22] Doley R, Zhou X, Kini M, Mackessy SP (2010). Snake venom phospholipase A2 enzymes. Handbook of venoms and toxins of reptiles.

[23] Braga MDM, Costa Martins AM, Amora DN, Beserra de Menezes D, Toyama MH, Toyama DO, Marangoni S, Alves CD, Ferreira Barbosa OS, de Sousa Alves R, Fonteles MC, Azul Monteiro HS (2008). Purification and biological effects of L-amino acid oxidase isolated from *Bothrops insularis* venom. Toxicon.

[24] Lee ML, Chung I, Fung SY, Kanthimathi MS, Tan SH (2014). Antiproliferative activity of king cobra (*Ophiophagus hannah*) venom L-amino acid oxidase. Basic Clin Pharmacol Toxicol.

[25] Fung SY, Lee ML, Tan NH (2015). Molecular mechanism of cell death induced by king cobra (*Ophiophagus hanna*) venom L-amino acid oxidase. Toxicon.

[26] Sharma RD, Katkar GD, Sundaram MS, Paul M, NaveenKumar SK, Swethakumar B, Hemshekhar M, Girish KS, Kemparaju K (2015). Oxidative stress-induced methemoglobinemia is the silent killerduring snakebite: a novel and strategic neutralization by melatonin. J Pineal Res.

[27] Ramírez GA, Fletcher PL, Possani DL (1990). Characterization of the venom from *Crotalus molossus nigrescens* Gloyd (Black tail rattlesnake): Isolation of two proteases. Toxicon.

[28] Escalante T, Shannon JD, da Silva AM M, Gutierrez JM, Fox JW (2006). Novel insights into capillary vessel basement membrane damage by snake venom hemorrhagic metalloproteinases: a biochemical and immunocyto-chemical study. Arch Biochem Biophys.

[29] Escalante T, Ortiz N, Rucavado A, Sánchez EF, Richardson M, Fox JW, Gutiérrez JM (2011). Role of Collagens and perlecan in microvascular stability: Exploring the mechanism of capillary vessel damage by snake venom metalloproteinases. PLoS ONE.

[30] Benseny-Cases N, Klementieva O, Cotte M, Ferrer I, Cladera J (2014). Microspectroscopy (μFTIR) reveals co-localization of lipid oxidation and amyloid plaques in human Alzheimer disease brains. Anal Chem.

[31] Vargas-Caraveo A, Castillo-Michel H, Mejia-Carmona G, Pérez-Ishiwara D, Cotte M, Martínez-Martínez A (2014). Preliminary studies of the effects of psychological stress on circulating lymphocytes analyzed by synchrotron radiation based-Fourier transform infrared microspectroscopy. Spectrochim Acta A Mol Biomol Spectrosc.

[32] Al Asmari A, Al Moutaery K, Manthari RA, Khan HA (2006). Time-course of lipid peroxidation in different organs of mice treated with *Echis pyramidum* snake venom. J Biochem Mol Toxicol.

